# Public Health Midwives as a Family Health Care Worker to Promote Vaginal Health: Evidence from a Community Study in Sri Lanka

**Published:** 2020-05

**Authors:** Ilankoon Mudiyanselage Prasanthi Sumudrika ILANKOON, Christine Sampatha Evangeline GOONEWARDENA, Rukshan Cleophas FERNANDOPULLE, Poruthotage Pradeep Rasika PERERA

**Affiliations:** 1. Department of Nursing and Midwifery, Faculty of Allied Health Sciences, University of Sri Jayewardenepura, Nugegoda, Sri Lanka; 2. Department of Community Medicine, Faculty of Medical Sciences, University of Sri Jayewardenepura, Nugegoda, Sri Lanka; 3. Department of Obstetrics and Gynecology, Faculty of Medical Sciences, University of Sri Jayewardenepura, Nugegoda, Sri Lanka; 4. Department of Biochemistry, Faculty of Medical Sciences, University of Sri Jayewardenepura, Nugegoda, Sri Lanka

## Dear Editor-in-Chief

Sri Lanka is a lower middle income country where the public health care is provided free of charge and is delivered through curative and preventive health services (1). The Public Health Midwives (PHMs) is the grass root level health care worker in the healthcare system of Sri Lanka (2). PHMs are recognized by the public as caring and skilled professionals (3) which is not only focusing on midwifery, but preventive health covering many other aspects (2).

Gynaecological complaints are frequent reasons for seeking primary healthcare (4) and among the most common complaints is vulvo-vaginal discharge which compromise the sexual and reproductive health of women (4). Lack of knowledge on vulvo-vaginal discharge leads to unhealthy practices among women at reproductive age. The PHMs can address this issue by educating women and promoting vaginal health.

Thus, this community based descriptive cross sectional study was aimed to assess the PHMs’ current level of knowledge on vulvo-vaginal discharge in order to educate community. The study was carried out in the Colombo District, Sri Lanka during January to December 2015. A self-developed, validated, pretested self-administered questionnaire was used for the data collection. Ethical clearance was obtained from the Ethics Review Committee of the Faculty of Medical Sciences, University of Sri Jayewardenepura, Sri Lanka (Ref No: 27/14). SPSS software version 16 was used in analysis.

A total of 308 PHMs participated and the mean age was 40.69 years (SD±10.72). Mean years of working as a PHM was 13.15 years (SD ± 10.01). In another Sri Lankan study, PHMs were middle-aged or older, most with more than 20 years of work experience (3). The place of health education for majority of PHMs were home visits (72.7%) and at clinics (66.2%). The most common topics used in their routine health education were pregnancy (254, 82.5%), family planning (246, 79.9%), breastfeeding (198, 64.3%) and early childhood development (161, 52.3%). Despite of their midwifery training, only half of them had received information on normal/abnormal vaginal discharge through other training programmes. But no one had received training related to reproductive tract morbidities after their basic training. The PHMs can be assigned for health promotion activities but the competency on a particular subject need to be concerned.

Mean knowledge score on vaginal discharge among the study participants was 53.6 (SD ± 12.43). Ten participant had a good level of knowledge (>75%) and 48.7% (n=150) had moderate level of knowledge (50%–75%). Nearly half of the study sample (48%, n= 148) had inadequate level of knowledge (<50%) on vaginal discharge.

The mean knowledge scores were significantly higher among those who had less than 40 years of age (p= 0.004) and work experience less than 15 years (p=0.002) to their counterparts in the present study. It is a proven fact that older age groups perform less in short-term memory, making it difficult for them to acquire and retrieve information (5). The PHMs who were trained on health promotion had a ‘good’ level of knowledge in contrast to the present study (6). The major areas that the PHM’s lacked knowledge in the present study were on reproductive tract infections and possible causes for pathological vulvo-vaginal discharge (Table 1).

**Table 1:** PHMs’ knowledge regarding Vaginal Discharge (n=308)

***Statements***	***True***	***False***	***Don’t Know***
A clear, non-offensive discharge that varies with the menstrual cycle is a normal physiological secretion.	295 (95.8)	10 (3.2)	3 (1)
Vaginal secretions vary with menstrual cycle.	261 (84.7)	29 (9.4)	18 (5.8)
The most common cause of vaginal discharge is Sexually transmitted infections (STIs).	90 (29.2)	185 (60.1)	33 (10.7)
Women aged between 15–49 years have a normal physiological vaginal secretion.	273 (88.6)	22 (7.1)	13 (4.2)
White or colored vaginal discharge may be a sign of infections.	229 (74.4)	49 (15.9)	30 (9.7)
Vaginal secretions can be increased during pregnancy.	269 (87.3)	15 (4.9)	24 (7.8)
Candida infection is a STI.	194 (63)	91 (29.5)	23 (7.5)
Candida causes vulval itch, soreness and thick white offensive discharge.	292 (94.8)	10 (3.2)	6 (1.9)

Specific areas of knowledge gaps on vulvovaginal discharge identified need to be strengthened by using a more comprehensive approach in order to empower PHMs as a family health care worker to conduct health education in the community level and to improve women’s health.
